# Clinical and genetic analysis in Chinese children with Alagille syndrome

**DOI:** 10.1186/s12887-022-03750-z

**Published:** 2022-11-29

**Authors:** Ying Chen, Mei Sun, Xu Teng

**Affiliations:** Department of Pediatric Gastroenterology, Shengjing Hospital of China Medical University, Shenyang, 110004 Liaoning China

**Keywords:** Alagille syndrome, clinical manifestation, Genetic analysis, China

## Abstract

**Background:**

Alagille syndrome (ALGS) is a multisystem disorder with variable clinical penetrance. The genes responsible for this disease are *JAGGED1 (JAG1)* and *NOTCH2*. Clinical data of this disease are limited in China. The purpose of this study was to enrich the present data of Chinese children with Alagille syndrome by summarizing the clinical characteristics and genetic variations of these cases.

**Case summary:**

From January 2011 to February 2022, 10 children were diagnosed with ALGS. The organs involved in ALGS were as follows: liver (10, 100%); heart (7, 70%); characteristic facial features (7, 70%); skeleton (4, 40%); brain (1,10%) and kidney (3, 30%). Four patients (40%) were small for gestational age. The main clinical manifestations were cholestasis, heart disease, and facial features. The median total bilirubin, direct bilirubin, and total bile acid levels were 138.75 μmol/L (normal, 3.4–20.5 μmol/L), 107.25 μmol/L (normal, 0–8.6 μmol/L), and 110.65 μmol/L (normal, 0.5–10.0 μmol/L), respectively. The median value of gamma-glutamyltranspeptidase was 223 U/L (normal, 9–64 U/L). Six (60%) children had hypercholesteremia. Eight different *JAG1* gene variations and one *NOTCH2* gene pathogenic variant in the 10 Chinese ALGS patients were identified.

**Conclusion:**

Cholestasis was the most common initial presenting symptom in Chinese ALGS pediatric patients. Pathogenic variants in *JAG1* and *NOTCH2* are the primary mutations in Chinese children with ALGS, but we had our own unique variant spectrum. ALGS should be considered for cholestasis in infants and young children, especially those with multiorgan abnormalities.

## Introduction

Alagille syndrome (ALGS) is a multisystem disease. The main organs involved in this disease are the liver, heart, eyes, skeleton, face, kidneys, and vasculature. With the development of molecular diagnostic technology, the incidence is likely 1 in 30,000 live births [1]. At present, there are no epidemiological data on this disease in China.

ALGS is an autosomal dominant disorder. Ninety-four percent of ALGS cases are due to pathogenic variants in the *JAG1* gene encoding the JAGGED1 protein, 1.5% are due to pathogenic variants in the *NOTCH2* gene, and 4.5% are due to unknown pathogenic genes [2]. In general, genetic confirmation is necessary because clinical presentation and disease severity are highly variable [3].

Although pathogenic variants causing ALGS have now been identified, the lack of genotype–phenotype correlations makes diagnosis of this disease difficult [4]. Some studies have reported the clinical and pathologic characteristics and gene variations of ALGS. Clinical data for Chinese ALGS patients are limited. We summarized the clinical features of ALGS in our hospital. Genetic pathogenic variants in Chinese children with ALGS were also analyzed. We hope that our work will improve our understanding of ALGS disease in China, expand the pathogenic gene variant spectrum of ALGS, and provide assistance for the diagnosis and treatment of children, as well as family genetic counseling and prenatal diagnosis.

## Materials and methods

The clinical data of patients diagnosed with ALGS in our hospital from January 2011 to February 2022 were retrospectively analyzed. Some patients initially presented to the local clinic for “jaundice”. They were transferred to our hospital soon after evaluation of their condition. To exclude extrahepatic disorders such as biliary atresia (BA) and infectious and metabolic disorders, we completed a series of tests, including blood ammonia, lactic acid and blood glucose monitoring, blood gas ion analysis, bacterial culture, viral serology, thyroid function and serum amino acid analysis. Urine specimens were reserved for routine examination, cytomegalovirus nucleic acid detection and organic acid analysis. Imaging examinations included liver and spleen ultrasonic and magnetic resonance cholangiopancreatography examinations.

ALGS should be suspected in individuals with histological observation of bile duct paucity (decreased bile duct-to-portal tract ratio) on liver biopsy and three of the following five major clinical features: cholestasis, cardiac defect (cardiac auscultation and echocardiography), skeletal abnormalities (chest radiograph), ophthalmologic abnormalities (subspecialty consultation and slit lamp examination under sedation), and characteristic facial features (triangular face with a broad forehead and pointed chin, bulbous tip of the nose, deeply set eyes, and hypertelorism) [5]. Most parents were reluctant to undergo liver biopsy because it is an invasive procedure. Liver biopsies were performed in only two cases in this study. All children had a heterozygous pathogenic variant in *JAG1* or *NOTCH2*, as identified by molecular genetic testing.

The data included demographic characteristics (e.g., age, sex, weight and length at birth, weight on admission), organs involved, liver function parameters [alanine transaminase (ALT), aspartate transaminase (AST), gamma-glutamyltranspeptidase (GGT), serum total bilirubin (TBil), direct bilirubin (DB), and total bile acid (TBA) levels], total cholesterol (TC) levels, and the molecular diagnosis of the genetic pathogenic variant.

### Pathogenic variant analysis

Blood samples were obtained from the children and their parents. Genomic DNA was extracted using a blood genomic DNA extraction kit (MyGenostics Co., Ltd., Beijing). Targeted regions were amplified, purified and sequenced by high-throughput sequencing with Nextseq 500 (Illumina, San Diego, California, USA). Sequencing was performed by Beijing MyGenostics Medicine Research Center Co. Ltd. (Beijing, China). The databases for MAF annotation include the 1,000 genomes, dbSNP, ESP, ExAC, and MyGenostics in-house MAFs databases. Protein product structure variations were predicted by the Provean, Sift, Polypen2_hdiv, Polypen2_hvar, Mutationtaster, M-Cap, and Revel software packages. As a prioritized pathogenicity annotation to the ACMG guidelines, the OMIM, HGMD, and ClinVar databases were used to evaluate the pathogenicity of each variant. The MaxEntScan, dbscSNV, and GTAG software packages were used to predict functional changes of variants on the splicing sites.

For statistical analysis, values are presented as the median, and categorical data are presented as numbers and percentages.

## Results

There were 10 children in this study (eight boys and two girls), with a median age of 2.5 months (age range, 1–72 months). The organs involved in ALGS were as follows: liver (10, 100%): cholestasis (8, 80%), hepatomegaly (7, 70%), and simple hepatic dysfunction (abnormally elevated transaminases, > 2 times the normal value) (2, 20%); heart (7, 70%): aortic dysplasia (1, 14.3%), aortic stenosis (1, 14.3%), pulmonary artery stenosis (3, 42.8%), atrial septal defect (1, 14.3%), ventricular septal defect (1, 14.3%); characteristic facial features (7, 70%); epileptic seizure (1, 10%); and butterfly vertebrae (4, 40%). Three children (30%) had kidney involvement. The renal ultrasound revealed rough renal parenchyma in 2 children and hydronephrosis of the left kidney in the other child. Routine urine examination showed no abnormalities. No children had ophthalmological abnormalities. Six children (60%) had hypercholesteremia [median value, 6.02 mmol/L (normal, 3.36–5.69 mmol/L)]. Four children (40%) were below the 10^th^ percentile in birth weight. The median total bilirubin, direct bilirubin, and total bile acid levels were 138.75 μmol/L (normal, 3.4–20.5 μmol/L), 107.25 μmol/L (normal, 0–8.6 μmol/L), and 110.65 μmol/L (normal, 0.5–10.0 μmol/L), respectively. The median value of gamma-glutamyltranspeptidase (GGT) in the 10 children was 223 U/L (normal, 9–64 U/L). One patient’s liver pathology showed intrahepatic bile duct paucity and giant cell hepatitis. The other patient’s liver pathology showed mild ductular proliferation, a few lymphocytes infiltrated the portal area, mild liver fibrosis, and no bile plugs (Tables 1 and 2).

**Table 1:** Demographic and clinical characteristics of 10 children with Alagilla syndrome. *N* = 10

Age (months)	2.5(1–72)
Gender (n, %)	
Male	8(80%)
Female	2(20%)
Clinical symptoms (n, %)	
Hepatic features	10(100%)
Cardiac features	7(70%)
Facial features	7(70%)
Ocular features	0(0)
Skeletal features	4(40%)
Renal features	3(30%)
Hypercholesteremia	6(60%)
Small for gestational age	4(40%)
Epileptic seizure	1 (10%)
Liver function	
Total bilirubin(umol/L) (normal, 3.4–20.5 umol/L),	138.75(6.2–183.6)
Direct bilirubin(umol/L) (normal, 0–8.6 umol/L)	107.25(2.4–148.3)
Total bile acid(umol/L) (normal, 0.5–10.0 umol/L)	110.65(6.7–481.2)
Gamma-glutamyltranspeptidase(U/L) (normal, 9–64 U/L)	223(34–1139)
Total cholesterol(mmol/L) (normal, 3.36–5.69 mmol/L)	6.02(3.64–7.65)
Genetic variation	
*JAG1* (n, %)	9(90%)
*NOTCH2* (n, %)	1(10%)

**Table 2:** Case series of 10 children with Alagille syndrome

**Patient**	Age(m**)/** Gender	Weight percentiles at birth	Organs involved	Liver function						TC (mmol/L) (3.36–5.69)	Liver biopsy	Genetic variation
				**ALT (U/L) (0–40)**	**AST (U/L) (5–34)**	**GGT (U/L) (9–64)**	**TBil (umol/l) (3.4–20.5)**	**DB (umol/l) (0–8.6)**	**TBA (umol/l) (0.5–10.0)**			
**1**	1/F	50^th^	cholestasis, hepatomegalyaortic dysplasia butterfly vertebraerenal parenchymal roughness	137	154	177	146.8	120.6	99.5	6.11	N	*NOTCH2*
**2**	2/M	< 3^rd^	cholestasis, hepatomegaly supravalvular aortic stenosis butterfly vertebrae facial features	88	211	1139	127.7	104.3	105.4	6.25	N	*JAG1*
**3**	2/M	< 3^rd^	cholestasis, hepatomegaly pulmonary artery stenosis facial features renal parenchymal roughness	439	576	34	181.1	141.0	123.8	4.25	intrahepatic bile duct paucity. giant cell hepatitis	*JAG1*
**4**	72/M	10-25^th^	hepatic dysfunction facial features butterfly vertebrae hydronephrosis of the left kidney	98	86	117	6.2	2.4	6.7	3.64	N	*JAG1*
**5**	4/M	< 3^rd^	cholestasis, hepatomegaly atrial septal defect facial features	54	117	613	160.7	135.3	481.2	7.48	N	*JAG1*
**6**	3/M	3-10^th^	cholestasis, hepatomegaly ventricular septal defect facial features epilepsy	253	347	106	130.7	110.2	135.5	4.05	N	*JAG1*
**7**	1/M	50^th^	cholestasis, hepatomegaly pulmonary artery stenosis	37	63	157	106.7	48.7	159.9	7.65	ductular proliferation. lymphocytic infiltration in portal area;mild fibrosis; no bile plugs	*JAG1*
**8**	3/F	50^th^	hepatic dysfunction facial features	552	250	746	18.1	13.7	32.4	6.05	N	*JAG1*
**9**	6/M	25-50^th^	cholestasis, hepatomegaly facial features	41	69	269	146.8	83.4	92.1	5.99	N	*JAG1*
**10**	1/M	50-75^th^	cholestasis, acholic stool Common bile duct dysplasia, pulmonary artery stenosis, butterfly vertebrae	217	230	625	183.6	148.3	115.9	4.41	N	*JAG1*

abbreviations’ footnote: *m* Months, *ALT* Alanine transaminase, *AST* Aspartate transaminase, *GGT* Gamma-glutamyltranspeptidase, *TBil* Serum total bilirubin, *DB* Direct bilirubin, *TBA* Total bile acid, *TC* Total cholesterol, *N* No liver biopsy was performed

The results of the genetic analyses are summarized in Table 3. Alterations in the *JAG1* gene were identified in nine patients, and the *NOTCH2* gene was sequenced in one patient. Eight distinct *JAG1* pathogenic variants were identified (Cases #2 and #8 had the same gene pathogenic variant). Of these, six were novel and included five nonsense pathogenic variants (55.6%), three frameshift pathogenic variants (33.3%), and one splicing pathogenic variant (11.1%). The pathogenic variants were located in the seven exons (6, 7, 11, 12, 14, 23 and 25) that constitute the coding region of the *JAG1* gene. The novel variation of *NOTCH2* was the nonsense pathogenic variant c.3928C > T in exon 24.

**Table 3:** Genetic traits of children with Alagille syndrome

**Patient**	**Gene**	**Exon**	**Genetic mutation information**	**mutation type**	**Homozygous**/**Heterozygous**	**Family results**	**ACMG Pathogenicity analysis**
1	*NOTCH2*	exon24	c.3928C > T p.Q1310X	nonsense	heterozygous	mother	Likely pathogenic
2	*JAG1*	exon23	c.2698C > T p.R900X	nonsense	heterozygous	spontaneous variant	pathogenic
3	*JAG1*	exon12	c.1464delC p.I488Mfs*10	frameshift	heterozygous	spontaneous variant	pathogenic
4	*JAG1*	exon25	c.3094_3095delAC p.T1032*fs*1	frameshift	heterozygous	spontaneous variant	pathogenic
5	*JAG1*	exon14	c.1853delA p.N618Tfs*125	frameshift	heterozygous	Spontaneous variant	pathogenic
6	*JAG1*	exon6	c.2080A > T p.K694X	nonsense	heterozygous	Spontaneous variant	pathogenic
7	*JAG1*	exon11	c.1395 + 3A > G splicing	splicing	heterozygous	mother	Likely pathogenic
8	*JAG1*	exon23	c.2698C > T p.R900X	nonsense	heterozygous	spontaneous variant	pathogenic
9	*JAG1*	exon12	c.1437 T > A p.Y479X	nonsense	heterozygous	Spontaneous variant	pahogenic
10	*JAG1*	exon7	c.960 T > G p.Y320X	nonsense	heterozygous	Spontaneous variant	Pathogenic

abbreviations’ footnote: *m* months, *ALT* Alanine transaminase, *AST* Aspartate transaminase, *GGT* Gamma-glutamyltranspeptidase, *TBil* Serum total bilirubin, *DB* Direct bilirubin, *TBA* Total bile acid, *TC* Total cholesterol, *N* No liver biopsy was perform

## Discussion

Cholestasis is a key clinical feature that is usually present in the first 3 months after birth [6]. In our retrospective study, the vast majority (8/10, 80%) of children were admitted to the hospital with cholestasis. The most conserved feature of ALGS is bile duct paucity. Liver histology typically reveals a reduction in the concentration of intrahepatic bile ducts (bile duct to portal tract ratio < 0.4) [7]. As few as 60% of patients < 6 months showed paucity, whereas it was commonly found in 95% of infants aged > 6 months [6, 8]. However, the paucity trend is uncertain, and controversy still exists. More clinical data are certainly needed.

Currently, there is a growing consensus that liver biopsy is not required if clinical biochemical evidence shows cholestasis and other characteristics of ALGS [8]. This tendency prevents pediatric patients from undergoing invasive liver biopsy for definitive diagnosis. In our study, liver biopsies were performed for only two cases, but this did not affect diagnosis. Typical pathology is helpful for the diagnosis of ALGS, but the detection of typical clinical manifestations and gene mutations has decreased clinician dependency on pathology.

The most common differential diagnosis of ALGS is BA. BA is characterized by significant small bile duct hyperplasia. Ductular hyperplasia may also occur in ALGS patients, but it is milder than BA [9, 10]. The result of the liver biopsy of Case #7 was mild ductular proliferation, a few lymphocytes around the portal area, minimal liver fibrosis and no bile plugs. Nevertheless, liver ultrasound did not recognize a triangular or band-like periportal echogenicity or contractile dysfunction of the gallbladder. Magnetic resonance cholangiopancreatography reported that the intra- and extrahepatic bile ducts were continuously visualized without dilatation, and the gallbladder was large. Case #10 showed acholic stool, and the liver ultrasound indicated a small gallbladder and contractile dysfunction. However, both children had cardiac problems. The butterfly vertebrae in Case #10 were typical. Combined with genetic testing, ALGS was confirmed.

Kamath et al. reported that serum bile salts can remain elevated even when hyperbilirubinemia has been resolved [11]. Case #8 in the study showed normal total and direct bilirubin, but total bile acid remained elevated. GGT is also commonly elevated. The median value of GGT in the ten children in this study was 223 U/L (normal, 9–64 U/L). Case #3 was a 2-month-old boy with a normal GGT level (34 U/L). In children with normal GGT levels, familial progressive cholestasis is first suspected. However, this child also had pulmonary artery stenosis and facial features associated with ALGS. A liver biopsy suggested typical intrahepatic bile duct paucity. His *JAG1* pathogenic variant was novel (c.1464delC). Functional validation of the gene is ongoing. There are only a few reports of normal GGT levels in ALGS [10]. We speculate that normal GGT levels may represent early-stage ALGS in children. As the disease progresses, GGT levels increase, resulting in a pathological state.

Several larger descriptive studies showed renal and vascular abnormalities in many patients. Renal symptoms were not uncommon in our cases and were seen in three children. Rough renal parenchyma or hydronephrosis of the left kidney was found by ultrasound. Epilepsy was a unique symptom of Case #6 and is rare in ALGS. The child underwent magnetic resonance imaging, magnetic resonance cerebral angiography and venography, and no abnormalities were found. Although we have no direct evidence, we suspect that extracranial or intracranial vascular abnormalities could be a cause.

Ocular abnormalities are among the main features in the initial diagnosis of ALGS. The most important ocular abnormality involves the anterior segment and is the posterior embryotoxon. Recognition of this distinctive finding may be helpful for early diagnosis [12]. Sedation or anesthesia is necessary for children who are unable to cooperate with eye examinations. All 10 children in our study were sedated with chloral hydrate with the consent of their families and underwent a slit lamp examination by a professional ophthalmologist. Surprisingly, none of the children in our study showed ocular symptoms. We think there may be two reasons. First, posterior embryotoxon is so rare that our ophthalmologists could not accurately identify it. Many childhood liver diseases have ocular symptoms. It is necessary to share clinical information between pediatric hepatologists and ophthalmologists [12]. The more meticulous and accurate clinical data are, the better the help that ophthalmologists can provide. Second, we hypothesize that this may be a feature of Chinese children with ALGS. However, more clinical cases are needed to confirm this.

More than 50% of children with ALGS have growth and development disorders [13]. For 10 patients, the birth weight and birth length were recorded. According to growth standard curves for the birth weight, length and head circumference of Chinese newborns of different gestations in 2020, the 10^th^ percentile with a birth weight lower than the reference value was defined as small for gestational age [14]. Four out of 10 children were small for gestational age and remained underweight before admission to our hospital. Growth and development disorders are caused by many factors. One study considered that they are not secondary to clinical manifestations of ALGS, such as heart defects, but have a potential mechanistic link with intrinsic genetic defects [15]. The JAG1 and Notch signaling pathways play a role in bone development, and inadequate caloric intake and malabsorption of fat and fat-soluble vitamins caused by cholestasis are involved [16]. El Moghazy et al. [17] found that the total bilirubin level was significantly increased in children with growth retardation. A cohort study also found a moderate negative correlation between total bilirubin and Z score of height and weight [15]. The severity of liver disease may be an important factor negatively influencing growth and development. The sample size of this study was small, so the correlation between low body weight and liver disease was not obvious.

In ALGS, 94–95% of patients have a heterozygous pathogenic variant in *JAG1*, and 1–2% of patients have a heterozygous pathogenic variant in *NOTCH2 *[18]. Pathogenic variants in the *JAG1* gene were identified in nine patients (90%). Of these, five were nonsense pathogenic variants (55.6%), three were frameshift pathogenic variants (33.3%), and one was a splicing pathogenic variant (11.1%). The only identified *NOTCH2* pathogenic variant was a nonsense variant. Importantly, most of these genetic pathogenic variants have not been reported, indicating that our cases may have a unique variant spectrum.

ALGS cases with *JAG1* pathogenic variants usually present with facial dysmorphism and heart involvement compared to those with *NOTCH2* variants [19, 20]. Renal abnormalities may be more common in cases with *NOTCH2* pathogenic variants [18]. However, many studies have reported no clear correlation between genotype and phenotype for this disease. Phenotypic variability was observed among patients with the same pathogenic variants [21]. This type of variability has been associated with liver disease, with clinical manifestations ranging from mild liver function abnormalities to severe cholestasis [15]. In the present study, Cases #2 and #8 had the same genetic pathogenic variant, but the clinical manifestations differed. This supports the variable phenotypic penetrance of ALGS. Some studies have suggested that this may be due to the existence of other genes as genetic modifiers, such as the Fringe protein family, including LFNG, RFN, and MFNG, which can alter NOTCH signaling by regulating glycosyltransferase activity. THBS2 has also been considered a potential genetic modifier [22, 23].

Ten children were given drugs to protect the liver and promote bile excretion after admission and were discharged after cholestasis was relieved. Case #7 presented with cholestasis again with coagulopathy (prolonged INR) 3 months after discharge. After symptomatic treatment, he was transferred to the Liver Transplantation Center for liver transplantation. The total bilirubin and total bile acid levels of the other nine children gradually returned to normal during follow-up. Long-term follow-up studies and the living status of ALGS children are incomplete in our center. In the future, we hope to establish a more comprehensive follow-up to observe whether different pathogenic gene variants are meaningful in the evolution of ALGS.

The system of graded diagnosis and treatment in China is not good enough. Almost all children with severe cholestasis will choose the best hospital for the first diagnosis. Through this study, we realized that the primary pediatricians’ cognition of ALGS is insufficient, which is why we wrote this manuscript.

The characteristic finding of our study was that pathogenic variants in *JAG1* and *NOTCH2* are the primary variants in Chinese children with ALGS, but we had our own unique variants spectrum. The other surprising finding was that none of the patients had any ocular symptoms. Whether this is a feature of Chinese children with ALGS is unknown. We need more cases to confirm this. ALGS should be considered for cholestasis in infants and young children, especially those with multiorgan abnormalities.

## Data Availability

The datasets generated and analyzed during the current study are not publicly available because the request to upload the data was not approved by the Ethics Committee of Shengjing Hospital of China Medical University but are available from the corresponding author upon reasonable request.
